# Editorial Overview on Emerging Thermal Food Processing Technologies

**DOI:** 10.3390/foods11111543

**Published:** 2022-05-24

**Authors:** Mohsen Gavahian, Mahsa Majzoobi, Asgar Farahnaky

**Affiliations:** 1 Department of Food Science, College of Agriculture, National Pingtung University of Science and Technology, No. 1, Shuefu Road, Neipu, Pingtung 91201, Taiwan; 2 Biosciences and Food Technology, School of Science, RMIT University, Bundoora West Campus, Plenty Road, Melbourne, VIC 3083, Australia; mahsa.majzoobi@rmit.edu.au (M.M.); asgar.farahnaky@rmit.edu.au (A.F.)

In many cases, thermal processing technologies are necessary to provide safe food products. However, the restrictions of traditional thermal processes (e.g., the use of autoclaves and other systems based on conduction and convection modes of heat transfer) challenge the expectations of 21st-century food industry and consumers. Therefore, new thermal technologies, such as ohmic, microwave, radiofrequency, and infrared technologies, have emerged to address these issues. These technologies are promising for enhancing product safety and quality as well as their capacity to be used for the extraction, evaporation, drying, and other common thermal practices in the food industry. In addition, the industry is striving for new insights, hence, more details on mechanisms underlying these processes are needed to improve the scalability of these thermal emerging processing technologies while maintaining the quality parameters of product.

In this sense, a Special Issue entitled “Emerging Non-Thermal Food Processing Technologies” was launched in *Foods* (MDPI) to provide a forum for researchers to communicate some of their most recent findings on the applications of emerging thermal technologies for food processing. A total of 11 manuscripts were submitted to this Special Issue from various countries, and 6 of these were accepted for publication based on the peer-review process.

Among the research papers published in this Special Issue, “Effect of Combination of Time and Temperature on Quality Characteristics of Sous Vide Chicken Breast” was investigated by a research team [1]. This paper showed that chicken breast treated with a two-step temperature (50 and 60 °C) had an improved texture, lower cooking loss, acceptable redness values, and reduced lipid oxidation as compared with the samples cooked at the one-step temperature of 60 °C. The two-step Sous Vide process explored in this paper showed to be able to provide similar inactivation values of *Clostridium perfringens* and *Listeria monocytogenes* to the conventional one-step Sous Vide process.

Ohmic heating, as one of the most interesting emerging thermal processing technologies, was explored in one of the accepted papers in this Special Issue, entitled “Application and Effects of Ohmic-Vacuum Combination Heating on the Quality Factors of Tomato Paste” [2]. These researchers combined ohmic heating and vacuum to concentrate the tomato paste at various processing conditions. It was reported that an “Ohmic–Vacuum Combination” can be considered a promising approach due to its rapid heating, high efficiency, low energy consumption, and preservation of nutritional value.

Microwave-Assisted Induction Heating, as a novel approach, was explored in a paper entitled “Effect of a Novel Microwave-Assisted Induction Heating (MAIH) Technology on the Quality of Prepackaged Asian Hard Clam (*Meretrix lusoria*)” [3]. This study revealed the optimum heating conditions for prepackaged hard clams and elaborated on the effects of processing parameters on some quality parameters of the product.

A manuscript on “Effects of Radio Frequency Heating on the Stability and Antioxidant Properties of Rice Bran” explored the applicability of Radio Frequency to inactivate lipase of rice bran that accelerates lipid oxidation leading to product deterioration [4]. This study provides promising outcomes regarding the potential of this emerging technology for rice bran stabilization.

Another study investigated “Preliminary Evaluation of a Novel Microwave-Assisted Induction Heating (MAIH) System on White Shrimp Cooking” [5]. In this study, fresh white shrimps were sealed in crystallized polyethylene terephthalate packages and processed by MAIH at various temperatures and times to assess their effects on some quality parameters of the product. The results showed this processing approach can eliminate the post-pollution issue that MAIH heated and sterilized foods present after packing.

In addition to the above-mentioned original research articles, an informative review article was published in this Special Issue which focused on microwave heating, as one of the most commonly used emerging technologies in commercial units and home appliances. This article is entitled “Mechanistic and Machine Learning Modeling of Microwave Heating Process in Domestic Ovens” [6]. This paper provided valuable information on mechanistic models with different geometric dimensions and physics/kinetics which simulated the process of microwave heating. It also discussed model implementation and validation approaches. In addition, the authors elaborated on the integration of machine learning and mechanistic models in improving microwave heating performance as well as pure machine learning models based on experimental data.

In general, it can be concluded that the emerging thermal food processing technologies are subject to fundamental and applied research in many countries, and the strong industry demand for employing new processing techniques highlights the need for further research in this area, in particular for addressing the well-reported upscaling hurdles.
